# Cell-Based Chemical Safety Assessment and Therapeutic Discovery Using Array-Based Sensors

**DOI:** 10.3390/ijms23073672

**Published:** 2022-03-27

**Authors:** Mingdi Jiang, Aritra Nath Chattopadhyay, Vincent M. Rotello

**Affiliations:** Department of Chemistry, University of Massachusetts Amherst, 710 North Pleasant Street, Amherst, MA 01003, USA; mingdijiang@umass.edu (M.J.); aritranathch@umass.edu (A.N.C.)

**Keywords:** cellular phenotypic response, array-based sensor, multichannel, chemical risk assessment, therapeutics discovery

## Abstract

Synthetic chemicals are widely used in food, agriculture, and medicine, making chemical safety assessments necessary for environmental exposure. In addition, the rapid determination of chemical drug efficacy and safety is a key step in therapeutic discoveries. Cell-based screening methods are non-invasive as compared with animal studies. Cellular phenotypic changes can also provide more sensitive indicators of chemical effects than conventional cell viability. Array-based cell sensors can be engineered to maximize sensitivity to changes in cell phenotypes, lowering the threshold for detecting cellular responses under external stimuli. Overall, array-based sensing can provide a robust strategy for both cell-based chemical risk assessments and therapeutics discovery.

## 1. Introduction

Synthetic chemicals are used in almost every aspect of daily life, making it critical to know their acute and long-term health effects [1,2,3]. Additionally, new synthetic chemicals are being developed regularly by the pharmaceutical [4], agricultural [5], cosmetics, [6] and other related industries. Each of these new chemicals needs to be evaluated for toxicity. Similarly, the ability to assess the efficacy and off-target effects of drugs is essential to their use [7,8].

Cell-based screening assays are important tools in drug discovery and risk assessments, providing a less expensive alternative to animal models [9]. Additionally, the use of cell models provides the ethical benefit of minimizing animal use and suffering [10]. The most common cell-based approach for chemical safety assessments is cell viability [11]. These approaches are effective for predicting cell death or major cellular dysfunctions arising from acute chemical exposure [12]. Long-term exposure to low doses of synthetic chemicals, however, can induce more subtle cellular responses which are responsible for chronic diseases, including metabolic [13], autoimmune [14], neurocognitive [15] and cardiovascular diseases [16]. Intracellular and extracellular biomarkers provide useful indicators for detecting cellular abnormality, with limits of detection at the range of micromolar to nanomolar levels [17,18]. However, recent studies have shown that chronic exposure to far lower levels of chemicals can induce cellular phenotypic responses [19]. Additionally, biomarker-based strategies are generally expensive and require the multi-step processing of cells, limiting their application in high-throughput detection [20].

Cellular phenotypic signatures have the potential to be more sensitive indicators of chemical effects than conventional cell viability and biomarker-based measurements [21]. Hypothesis-free array-based sensing platforms can be engineered to maximize sensitivity to early and subtle cellular phenotypic changes [22]. This design capability makes hypothesis-free sensor arrays potential tools for both high-throughput chemical safety assessments and as important tools for probing both efficacy and off-target effects for drug discovery [23].

In this review, we focus on the opportunities provided by array-based sensing platforms for chemical safety assessments and therapeutic discoveries. We will briefly outline the design of array-based sensing platforms. In the following sections, we will review recent studies where array-based sensing strategies have been used for chemical risk assessments and drug efficacy screening. Finally, we will offer some insights on future directions on developing array-based sensing platforms for chemical safety assessments and therapeutic discoveries.

## 2. Design of Array-Based Sensing Platforms

### 2.1. Design and Fabrication of Array-Based Sensing System

**Figure 1:**
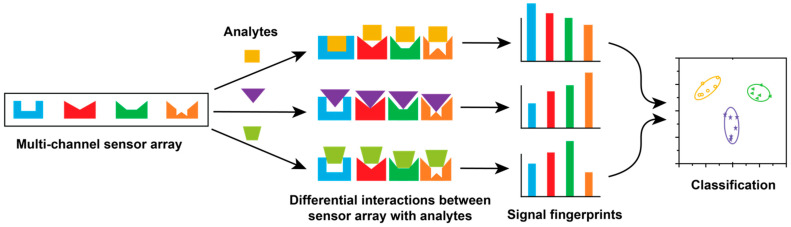
Schematic illustration of the array-based sensing platform. The multiple recognition elements in the sensor array interact with each analyte, generating distinguishable signal fingerprints, which can be classified using multivariate data analysis.

Sensors feature two connected processes: a recognition event, and a transduction process that creates a measurable output from this recognition event (Figure 1) [24,25]. For hypothesis-free array-based sensing systems, the recognition units should not be specific to any analyte; instead, desired cross-reactivity is generated between analytes towards recognition elements, forming distinct signal patterns [26]. Increasing the number of recognition elements and transduction elements can then be used to maximize sensitivity and improve the detection performance [27].

Array-based sensing systems can be created by using a wide range of synthetic elements [28,29,30,31]. Nanoparticles are one of the most commonly used sources for sensor array fabrication due to their ease of functionalization and large surface areas, providing surfaces for biomolecular recognition [32,33,34]. In addition, metallic nanoparticles are excellent fluorescent quenchers, providing distinguishable fluorescent signatures with a higher sensitivity that facilitates transduction [35]. Another promising material is synthetic polymers, which have high stability and scalability [36,37,38,39]. Both recognition elements and dyes can be added to a single polymer to reduce the sensor elements but increase the sensitivity [40]. Finally, synthetic small-molecule fluorescent compounds are also useful sources for developing sensor arrays due to their small size and high sensitivity towards targets [41,42].

### 2.2. Multivariate Data Analysis for Array-Based Sensing

The cross-reactivity of sensor arrays enables the generation of high-dimensional and high-content data [43]. The complexity of the signal data is readily amenable to multivariate data analysis strategies that reduce the dataset dimensionality and provide quantitation [44]. Use of these machine learning techniques helps in the classification and prediction of data, as well as facilitating interpretation [45]. Machine learning primarily has two types: unsupervised learning and supervised learning [46]. Unsupervised learning algorithms learn from unlabeled test data without classification. They directly identify commonalities in each new piece of data. Principle component analysis (PCA) and hierarchical clustering analysis (HCA) are two commonly used unsupervised methods [47,48]. Conversely, supervised methods, including linear discriminant analysis (LDA), have a set of training data to classify the known samples, giving a reference to identify unknown samples [49,50]. Both unsupervised and supervised learning methods play important roles in array-based sensing. For example, Shin et al. used array-based sensing with PCA to identify target volatile organic compounds in contaminated humid air [51]. Pan et al. used LDA and other machine learning methods to assist with surface-functionalized carbon dot sensor arrays for discriminating different types of proteins [52]. De et al. used different machine learning methods to analyze the sensitivity of cationic MoS2 and GFP conjugates for discriminating protein types in serum media and compared the classification accuracy of different methods [53]. Unsupervised learning methods such as PCA are important in identifying trends in large sets of data using statistical parameters. LDA, on the other hand, depends on a ‘training set’ of data to create a model which organizes data into defined classes based on the input from the user. Based on this trained algorithm, a secondary independent dataset can be tested and the success of the classification of the secondary data set provides an indication of the accuracy of the model. Supervised learning methods, therefore, offer a method of quantitation of unknown analytes based on input data and a more accurate prediction of these unknown analytes [54].

## 3. Applications of Array-Based Cell Sensing for Chemical Screening

Array-based sensors are engineered to maximize their sensitivity towards analytes, often identifying subtle changes in complex patterns. Array-based sensors are becoming important tools in a range of applications [55,56]. For example, they are widely applied to sense different chemical species for monitoring environmental conditions [57,58,59] and food quality [60]. Particularly, array-based sensing platforms are well suited to detect early and subtle changes in complex biosystems present in/on mammalian cells and bacteria [61,62,63,64,65]. Array-based cell sensing often employs interactions of sensors with cell surface components (phospholipids, proteins, and carbohydrates, etc.), which are different between cell types and states, making them excellent targets for rapidly assessing cell responses under environmental stimuli [66,67].

### 3.1. Array-Based Cell Sensing for Chemical Safety Assessment

#### 3.1.1. Environmental Safety Assessment

Synthetic chemicals are widely used in agriculture [68], food [69] and medicine [70], raising concerns and fears regarding potential risks to human health. Current chemical safety assessment approaches generally focus on acute health outcomes as the endpoints for assessing the risks posed by chemical agents [71]. This focus limits their application in the detection of early cellular responses following chemical exposure. Biomarkers provide a useful tool for detecting more subtle cellular abnormalities, but current biomarker-based strategies are generally expensive and need the multi-step processing of cells. Array-based sensing provides a more simple and rapid complementary method to detect subtle cellular phenotypic changes exposed to chemicals.

Living cells produce a large variety of metabolites [72,73]. Volatile compounds can provide valuable information about the physiological and metabolic state of cells [74,75]. Early studies using array-based sensing explored cellular volatile organic compounds (VOCs). Aldo et al. designed a metal–oxide semiconductor gas-sensor array to detect the changes in cell VOC profiles in response to the presence of chemical compounds [76]. This sensing was achieved through changes in electrical resistance resulting from the redox interactions of volatile compounds with sensor-surface-absorbed oxygen.

**Figure 2:**
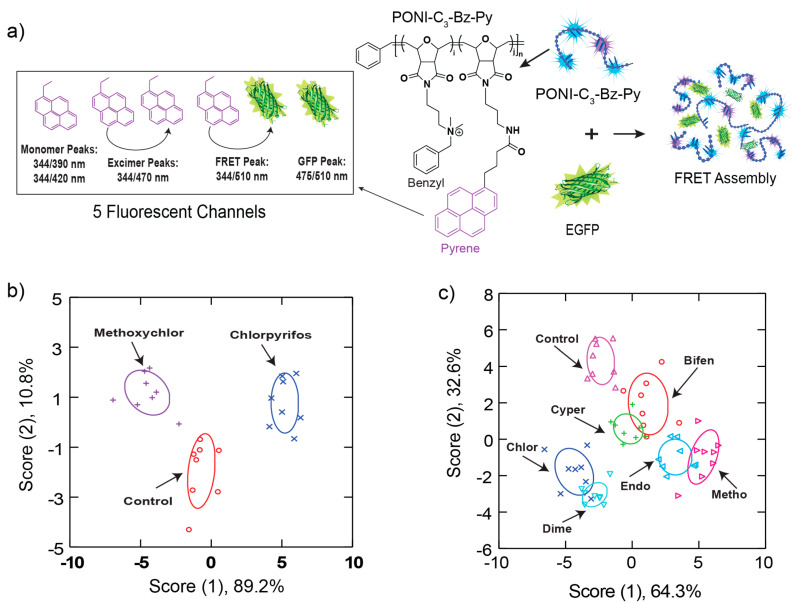
FRET-based sensor array for detecting cellular responses in macrophages induced by femtomolar level of pesticides. (**a**) The FRET-based sensor array was fabricated through a supramolecular assembly of PONI-C_3_-Bz-Py with EGFP, generating five fluorescence channels through fluorescence and FRET changes between the polymer and EGFP. (**b**) LDA classification of fluorescence responses from RAW 264.7 cells under methoxychlor or chlorpyrifos exposure (n = 8). (**c**) LDA classification of fluorescence responses from RAW 264.7 cells exposed to three classes of pesticides (n = 8). Reproduced with permission from [78]. Copyright 2022 Royal Society of Chemistry.

Pesticides are one of the most prevalent sources of chemical exposure due to their wide use in the food and agriculture industries [77]. Our group developed a multi-channel array-based sensing platform capable of detecting the effects of femtomolar levels of common pesticides on macrophages [78]. This system used a polymer–protein supramolecular assembly to generate a scalable sensor array platform. The sensor array was composed of a cationic benzylammonium-functionalized cationic poly(oxanorborneneimide) random copolymer conjugated with pyrene dye (PONI-C_3_-Bz-Py), electrostatically bound to anionic enhanced green fluorescent protein (EGFP). The benzyl group provides differential interactions with cell surface functionalities, resulting in changes in Förster resonance energy transfer (FRET) upon interactions of the sensor with cells. Additionally, the pyrene moiety displays an ensemble of monomeric fluorescence emission peaks and an excimer peak. Therefore, five fluorescent channels are generated in a single well (Figure 2a). The FRET-based nanosensor array detected and discriminated phenotypic changes in macrophages after 24 h exposure to femtomolar concentrations (10^−14^ M) of two common pesticides, chlorpyrifos and methoxychlor, with 96% correct classification and 96% accurate unknown identification (Figure 2b).In addition, this system was able to differentiate between different pesticide-induced phenotypes to classify pesticide class (Figure 2c), which confirmed the high sensitivity of array-based sensing for observing the effects of environmental chemicals on human health. Moreover, we also performed two widely used cytotoxicity assays (Alamar Blue assay and Trypan Blue exclusion assay) and a reactive oxygen species (ROS) detection assay to determine the effects of pesticides on RAW 267.4 cells at the 10^−14^ M concentration. No significant cell response was detected from these methods, further indicating that cellular phenotypic changes provide a more sensitive indicator of chemical effects than conventional cell viability, as well as the high promise of array-based sensing in drug discovery and diagnostics.

**Figure 3:**
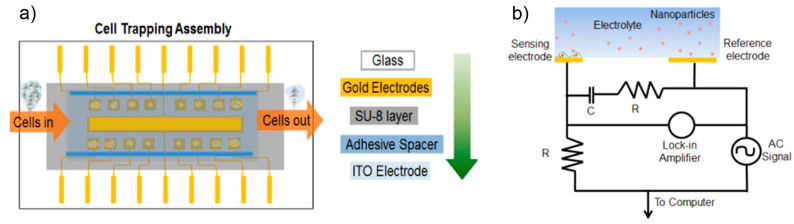
Cells-on-chip assembly. (**a**) Microelectromechanical system assembly for the dielectrophoretic trapping of cells; glass wafer is the base, gold sensing electrodes are under the SU-8 microwell pattern, spacer is used to hold the top ITO electrode. (**b**) Electronic cell impedance sensing circuit representing a single sensing electrode for simplicity. Reproduced with permission from [83]. Copyright 2016 American Chemical Society.

Nanomaterials are widely used in drug delivery [79], cell imaging [80], and consumer product development [81], leading to increased human contact. There are several cell-based approaches to study nanotoxicity using simple outputs [82]. Li et al. presented a microelectromechanical-system-based sensor array system to highlight the cell kinetics behavior of small-cell colonies of PC12 cells under exposure to NPs with different compositions [83]. The sensor array was fabricated using different sizes of microwells to hold different numbers of cells, and the cell responses under different NPs exposure were measured with a microelectromechanical system (MEMS) (Figure 3). The MEMS was fabricated with two different electrodes, an indium tin oxide (ITO) electrode and gold electrode, to generate dielectrophoresis (DEP) from a non-uniform dielectric field. DEP can manipulate the movement of particles by a trapping force when the particles and surrounding medium have different polarizabilities, offering a rapid and label-free toxicity detection method with high reproducibility. In this system, the cell impedance response to NPs was dependent on major changes in cell morphology and cell attachment.

**Figure 4:**
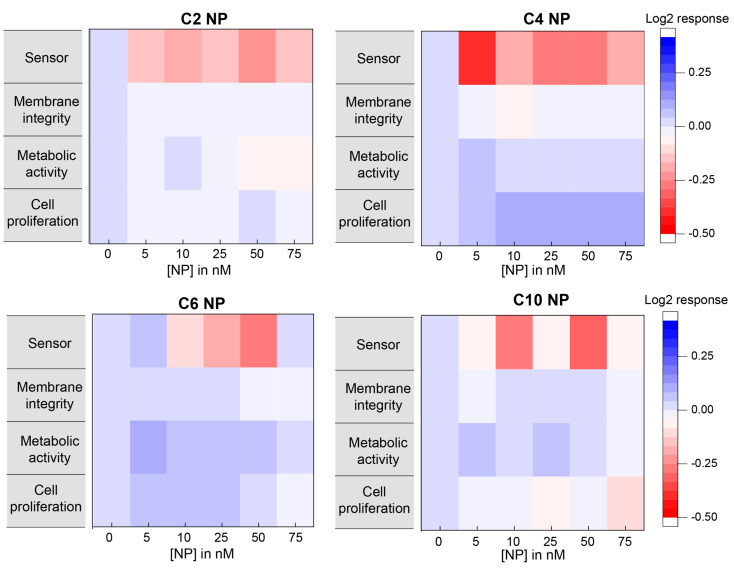
Comparison of MCF10A cellular responses after NPs exposure, as detected by the array-based nanosensor and standard cytotoxicity assays. The cellular responses from the nanosensor showed strong signals when exposed to C2–C10 NPs, whereas weak or null responses were obtained from other cytotoxicity methods. Reproduced with permission from [85]. Copyright 2020 Wiley-VCH.

Our lab created a hypothesis-free nanosensor through the electrostatic complexation of cationic gold nanoparticles (AuNPs) with anionic enhanced green fluorescent protein (EGFP). The fluorescence of EGFP can be quenched by AuNP and restored by the competitive interactions of AuNPs and biomacromolecular analytes. The multivalency of the nanoparticle provides high sensitivity, and fluorogenesis of the EGFP generates a robust fluorescent pattern. This sensor was initially used to discriminate metastatic cells and tissues [84]. The sensitivity displayed in these studies suggested that this platform could be used for the detection of cell phenotypes arising from nanoparticle exposure [85]. We determined the effects of ultra-low concentrations of a library of cationic nanoparticles with varying degrees of hydrophobicity (C2, C4, C6 and C10) on the non-malignant human mammary epithelial cell line MCF10A. In addition, we compared the sensing results with three commonly used cytotoxicity assays, Trypan Blue exclusion assay, Alamar Blue assay and DNA-staining Hoechst dye, which were used to evaluate cell membrane integrity, mitochondrial metabolism and cell proliferation, respectively. The nanosensor was readily able to detect phenotypic changes, whereas no response was observed using traditional cytotoxicity assays (Figure 4). Similarly, the AuNP-EGFP nanosensor was used to detect the estrogenic activity of low doses of endocrine-disrupting chemicals (EDCs) and their mixtures on MCF-7 cells [86].

#### 3.1.2. Therapeutics Safety Assessment

Toxicology plays an important role in drug development for evaluating the risk of potential drug candidates on human health [87]. For example, medications can cause acute kidney injury [88]. However, the complexity and diversity of various nephrotoxic mechanisms make risk assessments of nephrotoxic drugs challenging. Recently, Tian et al. constructed an array-based sensor using cationic polydopamine-polyethyleneimine (PDA-PEI) and three anionic quantum dots (QD515: CdSe/ZnS QD modified with 3-mercaptopropionic acid; QD580: CdSe/ZnS QD modified with PEG-COOH; QD640: CdSe/ZnS QD modified with l-cysteine) to classify nephrotoxic drug mechanisms based on the fluorescence changes arising from changes in cell surface phenotypes induced by multiple nephrotoxic drugs [89]. PDA-PEI is an effective quencher, and the QDs have a wide absorption and narrow emission, allowing multiple emission channels with a single excitation wavelength [90]. A total of 50 nephrotoxic drug from 7 classes were incubated with HK-2 cells at a concentration of IC_50_ for 24 h. The array-based sensor generated a unique fluorescent fingerprint for each class of drug-induced cell injury, and 50 drugs were separated into 7 clusters using both PCA and LDA, corresponding to 7 classes of drugs. These clusters were classified with 100% accuracy, and each cluster had an individual fluorescence signature trend over time.

### 3.2. Array-Based Cell Sensing for Therapeutics Discovery

The high-throughput screening of therapeutic efficacy and mechanism of drug candidates accelerates the discovery of new therapeutics [91]. Conventional screening methods, including screening genomic [92], transcriptional [93] and metabonomic [94] signatures, are time-consuming and require specialized equipment. The array-based sensing of cell surface phenotype signatures provides new directions for high-throughput and high-content screening (HT-HCS) methods for drug discovery.

**Figure 5:**
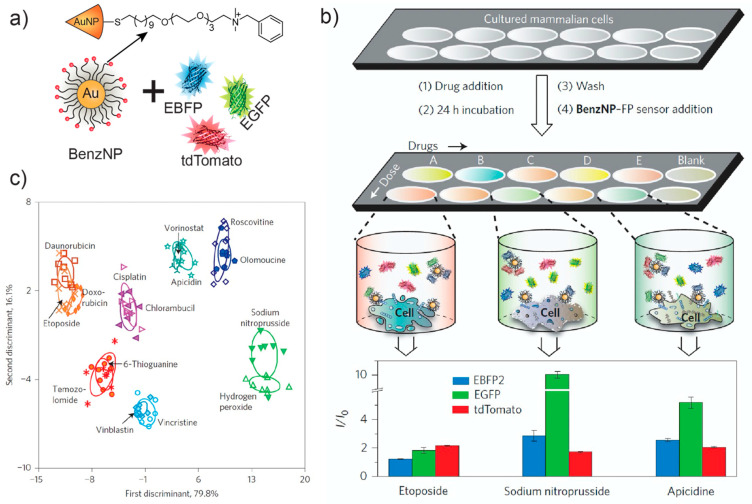
BenzNP-FPs array-based sensor for identifying cancer drug mechanisms. (**a**) Schematic illustration of BenzNP-FPs nanosensor array fabrication. (**b**) Workflow for chemotherapeutics screening using the BenzNP-FPs nanosensor array. (**c**) LDA classification of fluorescence responses from different drug mechanisms. Reproduced with permission from [95]. Copyright 2014 Springer Nature.

We developed a rapid multichannel sensor platform capable of profiling the mechanism of chemotherapeutic drugs in minutes [95]. This sensor uses a three-channel fluorescent protein (FP) platform analogous to the previously discussed EGFP systems [85,86]. In this study, the authors complexed a cationic AuNP with three different anionic FPs, EGFP, enhanced blue fluorescent protein (EBFP) and tandem dimer Tomato (tdTomato) (Figure 5). The nanosensor was used to screen 15 chemotherapeutics with different known molecular mechanisms to generate a training set of fluorescence fingerprints using LDA. The overlap of drugs with similar mechanisms and the separation of apoptotic and necrotic groups demonstrates the ability of the sensor to detect broader classes of cell death mechanisms. Significantly, the nanosensor can also predict unknown mechanisms and determine mechanistic correlations between individual drugs and their combinations. This identification was quantifiable through the use of Mahalanobis distances, a key advantage of LDA-based clustering [96]. In more recent work, this hypothesis-free AuNP-FPs sensor platform was used to identify nanoparticles capable of efficiently differentiating cancer stem cells (CSCs) into new phenotypes that are more susceptible towards traditional chemotherapeutics [97]. The susceptible phenotype had increased ROS levels and had synergistic effects with a metabolic inhibitor, 2DG on CSCs.

**Figure 6:**
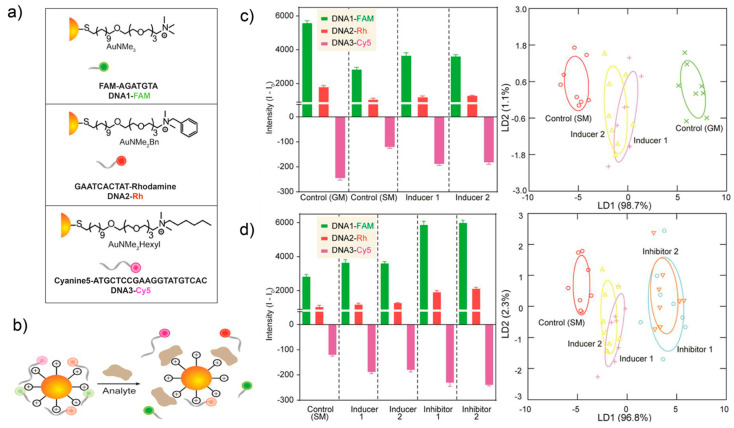
Multichannel DNA sensor array for detecting pharmacological effectors of catabolic processes. (**a**) The multi-channel sensor was created by complexing three different cationic AuNPs with different ssDNA sequences bearing distinguishable fluorescent signatures. (**b**) Competitive interaction between the quenched AuNP-DNA and the cell surface components creates a unique fluorescence pattern. (**c**) Sensor array generated different fluorescent fingerprints against the treatment of autophagy modulators, and the pharmacological inducers of autophagy were separated in LDA plots. (**d**) Sensor array generated distinguishable fluorescent fingerprints between cells treated with autophagy inhibitors and inducers, and the accuracy of discrimination under LDA was 98%. Reproduced with permission from [99]. Copyright 2019 American Chemical Society.

Single-stranded DNA (ssDNA) can be readily chemically synthesized to generate a large library, making these materials attractive motifs for sensing [98]. Agasti et al. complexed three cationic surface-functionalized AuNPs with different fluorophore-labeled ssDNA strands to form a robust multichannel array-based sensing platform [99]. Cells with different states were lysed to extract the total protein components. Proteins vary in size and possess their own signature of surface amino acid residues; therefore, they generate unique interactions with cationic AuNPs. The fluorescence of ssDNA was quenched by AuNPs via surface binding, but regenerated the fluorescence response when the lysate competitively interacted with AuNP, achieving the discrimination of cells based on their entire proteome signatures. The ability of this DNA-based multichannel sensor array to rapidly identify cell states encouraged authors to determine small-molecule autophagy modulator-induced global cellular state alterations, using LDA to assess the fluorescence signatures (Figure 6). The high accuracy of discrimination between inducers, inhibitors and control (98%) further demonstrated the excellent capability of the multichannel sensing system for high-throughput drug screening.

**Figure 7:**
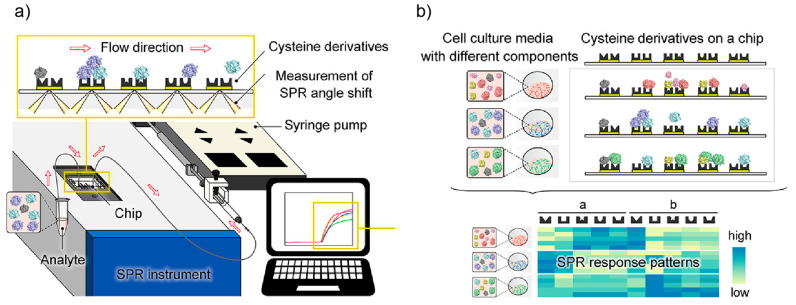
A multichannel SPR chip with immobilized cysteine derivatives for cell characterization. (**a**) Simultaneous acquisition of the SPR sensorgrams reflecting the interactions between cysteine derivatives on a chip and cell-secreted molecules. (**b**) Unique SPR response patterns resulting from cross-reactive interactions between cysteine derivatives and cell-secreted molecules. Reproduced with permission from [101]. Copyright 2020 American Chemical Society.

The combination of microfluidics with functional nanomaterials facilitates the rapid and sensitive detection of various bioanalytes [100]. Kurita et al. reported an array-based cell sensing strategy based on a multichannel surface plasmon resonance (SPR) chip, in which five cysteine derivatives with different structures were immobilized on Au films [101] (Figure 7a). When cells flowed into the chip, cell-secreted molecules interacted nonspecifically with cysteine derivatives, generating five unique SPR sensorgrams (Figure 7b). An automatic statistical program was built to acquire kinetic parameters from the SPR sensorgrams. The patterns of SPR responses were described as coefficients a and b for each probe, and curve fitting was carried out using R software. This microfluidic-based sensor array successfully identified different cell lines with 100% accuracy, with results mirrored in the testing of a model therapeutic, tamoxifen citrate (TAM). The multichannel microfluidic device allowed the on-site and real-time evaluation of cultured cells under external stimuli with high efficiency and accuracy.

## 4. Conclusions and Future Perspectives

The increase in synthetic chemical production and drug diversification greatly increases the need for new tools for chemical risk assessment. Cell-based screening assays are important tools in chemical risk assessments and drug discovery. Hypothesis-free array-based sensing platforms with cross-reactive properties have unique capabilities for discriminating cellular responses under external stimuli, enabling the achievement of high-throughput chemical safety assessments and therapeutic discoveries as compared with conventional cell viability and biomarker-based strategies. Significantly, the high sensitivity of array-based sensing enables the detection of more subtle cellular phenotypic changes under ultra-low doses of chemical exposure, facilitating the safer use of synthetic chemicals and the discovery of new therapeutic chemicals.

Array-based sensing systems have the potential to improve through the fabrication of more selective recognition elements and more sensitive transduction elements, as well as improving statistical analyses. In the near future, it will be important to combine the opportunities provided by hypothesis-free array-based sensing with the mechanistic understanding that biomarkers offer. These ‘hybrid’ platforms will allow for the better design of sensor arrays and better biomarker discovery for early chemical exposure diagnosis and therapy.

In summary, array-based sensing provides a promising method for detecting subtle cellular phenotypic changes under chemical exposure, which enable the early identification of ultra-doses of chemical-induced cellular responses. By combining sensor arrays with cellular biomarker discovery, array-based sensing will become a more robust and efficient tool for chemical risk assessments and drug candidate screening.

## Data Availability

Not applicable.

## References

[1] Barreiro M.F., Morales P., Ferreira I.C.F.R. (2014). Adding Molecules to Food, Pros and Cons: A Review on Synthetic and Natural Food Additives. Compr. Rev. Food Sci. Food Saf..

[2] Maitz M.F. (2015). Applications of Synthetic Polymers in Clinical Medicine. Biosurf. Biotribol..

[3] Bernhardt E.S., Rosi E.J., Gessner M.O. (2017). Synthetic Chemicals as Agents of Global Change. Front. Ecol. Environ..

[4] Campos K.R., Coleman P.J., Alvarez J.C., Dreher S.D., Garbaccio R.M., Terrett N.K., Tillyer R.D., Truppo M.D., Parmee E.R. (2019). The Importance of Synthetic Chemistry in the Pharmaceutical Industry. Science.

[5] Bhatt P., Gangola S., Bhandari G., Zhang W., Maithani D., Mishra S., Chen S. (2021). New Insights into the Degradation of Synthetic Pollutants in Contaminated Environments. Chemosphere.

[6] Duis K., Junker T., Coors A. (2021). Environmental Fate and Effects of Water—Soluble Synthetic Organic Polymers Used in Cosmetic Products. Environ. Sci. Eur..

[7] Docking M. (2021). Recent Advances on Small Molecule Medicinal Chemistry to Treat Human Diseases. Curr. Top. Med. Chem..

[8] Monge P., Bretschneider A., Guldbrandsen D., Chandrawati R., Zelikin A.N. (2021). Synthetic Chemical Ligands and Cognate Antibodies for Biorthogonal Drug Targeting and Cell Engineering. Adv. Drug Deliv. Rev..

[9] Jaroch K., Jaroch A., Bojko B. (2018). Cell Cultures in Drug Discovery and Development: The Need of Reliable in Vitro-in Vivo Extrapolation for Pharmacodynamics and Pharmacokinetics Assessment. J. Pharm. Biomed. Anal..

[10] Kim T.W., Che J.H., Yun J.W. (2019). Use of Stem Cells as Alternative Methods to Animal Experimentation in Predictive Toxicology. Regul. Toxicol. Pharmacol..

[11] Binh T., Cho S., Min J. (2013). Hydrogel-Based Diffusion Chip with Electric Cell-Substrate Impedance Sensing (ECIS) Integration for Cell Viability Assay and Drug Toxicity Screening. Biosens. Bioelectron..

[12] Meissner R., Eker B., Kasi H., Bertsch A., Renaud P. (2011). Distinguishing Drug-Induced Minor Morphological Changes from Major Cellular Damage via Label-Free Impedimetric Toxicity Screening. Lab Chip.

[13] Mesnage R., Teixeira M., Mandrioli D., Falcioni L., Ibragim M., Ducarmon Q.R., Zwittink R.D., Amiel C., Panoff J.M., Bourne E. (2021). Multi-Omics Phenotyping of the Gut-Liver Axis Reveals Metabolic Perturbations from a Low-Dose Pesticide Mixture in Rats. Commun. Biol..

[14] Hirai S., Naito M., Kuramasu M., Ogawa Y., Terayama H., Qu N., Hatayama N., Hayashi S., Itoh M. (2015). Low-Dose Exposure to Di-(2-Ethylhexyl) Phthalate (DEHP) Increases Susceptibility to Testicular Autoimmunity in Mice. Reprod. Biol..

[15] Lam H.S., Kwok K.M., Chan P.H.Y., So H.K., Li A.M., Ng P.C., Fok T.F. (2013). Long Term Neurocognitive Impact of Low Dose Prenatal Methylmercury Exposure in Hong Kong. Environ. Int..

[16] Klint H., Lejonklou M.H., Karimullina E., Rönn M., Lind L., Lind P.M., Brittebo E. (2017). Low-Dose Exposure to Bisphenol A in Combination with Fructose Increases Expression of Genes Regulating Angiogenesis and Vascular Tone in Juvenile Fischer 344 Rat Cardiac Tissue. Ups. J. Med. Sci..

[17] Dhandapani M., Goldman A. (2017). Preclinical Cancer Models and Biomarkers for Drug Development: New Technologies and Emerging Tools. J. Mol. Biomark. Diagn..

[18] Pappa A., Papadimitriou-tsantarliotou A., Kaloyianni M., Kastrinaki G., Dailianis S., Lambropoulou D.A., Christodoulou E., Kyzas G.Z., Bikiaris D.N. (2021). Insights into the Toxicity of Biomaterials Microparticles with a Combination of Cellular and Oxidative Biomarkers. J. Hazard. Mater..

[19] Latchney S.E., Majewska A.K. (2021). Persistent Organic Pollutants at the Synapse: Shared Phenotypes and Converging Mechanisms of Developmental Neurotoxicity. Dev. Neurobiol..

[20] Rusling J.F., Kumar C.V., Gutkind J.S., Patel V. (2010). Measurement of Biomarker Proteins for Point-of-Care Early Detection and Monitoring of Cancer. Analyst.

[21] Zheng W., Thorne N., Mckew J.C. (2013). Phenotypic Screens as a Renewed Approach for Drug Discovery. Drug Discov. Today.

[22] Main M.J., Zhang A.X. (2020). Advances in Cellular Target Engagement and Target Deconvolution. SLAS Discov..

[23] Geng Y., Peveler W.J., Rotello V.M. (2020). Array-Based “Chemical Nose” Sensing in Diagnostics and Drug Discovery. Angew. Chem. Int. Ed. Engl..

[24] Han K., Liang Z., Zhou N. (2010). Design Strategies for Aptamer-Based Biosensors. Sensors.

[25] Gil B., Akingbade O.E., Guo X., Gonzalez-macia L., Crone M.A., Cameron L.P., Freemont P., Choy K., Güder F., Yeatman E. (2022). Multiplexed Immunosensors for Point-of-Care Diagnostic Applications. Biosens. Bioelectron..

[26] Peveler W.J., Yazdani M., Rotello V.M. (2016). Selectivity and Specificity: Pros and Cons in Sensing. ACS Sens..

[27] Al Sulaiman D., Shapiro S.J., Gomez-Marquez J., Doyle P.S. (2021). High-Resolution Patterning of Hydrogel Sensing Motifs within Fibrous Substrates for Sensitive and Multiplexed Detection of Biomarkers. ACS Sens..

[28] Escobedo C. (2013). On-Chip Nanohole Array Based Sensing: A Review. Lab Chip.

[29] He Y., He X., Liu X., Gao L., Cui H. (2014). Dynamically Tunable Chemiluminescence of Luminol-Functionalized Silver Nanoparticles and Its Application to Protein Sensing Arrays. Anal. Chem..

[30] Feng F., Zheng J., Qin P., Han T., Zhao D. (2017). A Novel Quartz Crystal Microbalance Sensor Array Based on Molecular Imprinted Polymers for Simultaneous Detection of Clenbuterol and Its Metabolites. Talanta.

[31] Jing W., Lu Y., Yang G., Wang F., He L., Liu Y. (2017). Fluorescence Sensor Array Based on Amino Acids-Modulating Quantum Dots for the Discrimination of Metal Ions. Anal. Chim. Acta.

[32] De M., You C.C., Srivastava S., Rotello V.M. (2007). Biomimetic Interactions of Proteins with Functionalized Nanoparticles: A Thermodynamic Study. J. Am. Chem. Soc..

[33] Behera P., Mohanty A., De M. (2020). Functionalized Fluorescent Nanodots for Discrimination of Nitroaromatic Compounds. ACS Appl. Nano Mater..

[34] Sun J., Lu Y., He L., Pang J., Yang F., Liu Y. (2020). Colorimetric Sensor Array Based on Gold Nanoparticles: Design Principles and Recent Advances. Trends Anal. Chem..

[35] Yan J., Smith J.E., Wang K., He X., Wang L., Tan W. (2007). Dye-Doped Nanoparticles for Bioanalysis. Nano Today.

[36] Lee J.D., Greene N.T., Rushton G.T., Shimizu K.D., Hong J.I. (2005). Carbohydrate Recognition by Porphyrin-Based Molecularly Imprinted Polymers. Org. Lett..

[37] Bajaj A., Miranda O.R., Phillips R., Kim I., Jerry D.J., Bunz U.H.F., Rotello V.M. (2010). Array-Based Sensing of Normal, Cancerous, and Metastatic Cells Using Conjugated Fluorescent Polymers. J. Am. Chem. Soc..

[38] Alberti G., Zanoni C., Losi V., Magnaghi L.R. (2021). Current Trends in Polymer Based Sensors. Chemosensors.

[39] Stephenson C.J., Shimizu K.D. (2007). Colorimetric and Fluorometric Molecularly Imprinted Polymer Sensors and Binding Assays. Polym. Int..

[40] Ngernpimai S., Geng Y., Makabenta J.M., Landis R.F., Keshri P., Gupta A., Li C.H., Chompoosor A., Rotello V.M. (2019). Rapid Identification of Biofilms Using a Robust Multichannel Polymer Sensor Array. ACS Appl. Mater. Interfaces.

[41] Discenza D.J., Lynch J., Miller J., Verderame M., Levine M. (2017). Detection of Organochlorine Pesticides in Contaminated Marine Environments via Cyclodextrin-Promoted Fluorescence Modulation. ACS Omega.

[42] Bai X., Ng K.K., Hu J.J., Ye S., Yang D. (2019). Small-Molecule-Based Fluorescent Sensors for Selective Detection of Reactive Oxygen Species in Biological Systems. Annu. Rev. Biochem..

[43] Liu Y. (2012). Development of Cross-Reactive Sensors Array: Practical Approach for Ion Detection in Aqueous Media. Ph.D. Thesis.

[44] Fitzgerald J.E., Shen J. (2019). Spectroscopic Sensor Array for Organic Volatiles. Sensors.

[45] Spratt H., Ju H., Brasier A.R. (2013). A Structured Approach to Predictive Modeling of A Two-Class Problem Using Multidimensional Data Sets. Methods.

[46] Stewart S., Ivy M.A., Anslyn E.V. (2014). The Use of Principal Component Analysis and Discriminant Analysis in Differential Sensing Routines. Chem. Soc. Rev..

[47] Granato D., Santos J.S., Escher G.B., Ferreira B.L., Maggio R.M. (2018). Use of Principal Component Analysis (PCA) and Hierarchical Cluster Analysis (HCA) for Multivariate Association between Bioactive Compounds and Functional Properties in Foods: A Critical Perspective. Trends Food Sci. Technol..

[48] Crook A.A., Zamora-olivares D., Bhinderwala F., Woods J., Winkler M., Rivera S., Shannon C.E., Wagner H.R., Zhuang D.L., Lynch J.E. (2021). Combination of Two Analytical Techniques Improves Wine Classification by Vineyard, Region, and Vintage. Food Chem..

[49] Behera P., De M. (2019). Nano-Graphene Oxide Based Multichannel Sensor Arrays towards Sensing of Protein Mixtures. Chem. Asian J..

[50] Herrig R., Moriwaki T., Falcioni R., Pattaro M., Vollmann A., Carlos A., Junior S., Camargos W., Rafael M. (2020). Remote Sensing Applications: Society and Environment Hyperspectral Reflectance Imaging to Classify Lettuce Varieties by Optimum Selected Wavelengths and Linear Discriminant Analysis. Remote Sens. Appl. Soc. Environ..

[51] Itoh T., Akamatsu T., Tsuruta A., Shin W. (2017). Selective Detection of Target Volatile Organic Compounds in Contaminated Humid Air Using a Sensor Array with Principal Component Analysis. Sensors.

[52] Pandit S., Banerjee T., Srivastava I., Nie S., Pan D. (2019). Machine Learning-Assisted Array-Based Biomolecular Sensing Using Surface-Functionalized Carbon Dots. ACS Sens..

[53] Behera P., Singh K.K., Pandit S., Saha D., Saini D.K., De M. (2021). Machine Learning-Assisted Array-Based Detection of Proteins in Serum Using Functionalized MoS2 Nanosheets and Green Fluorescent Protein Conjugates. ACS Appl. Nano Mater..

[54] Delen D. (2010). A Comparative Analysis of Machine Learning Techniques for Student Retention Management. Decis. Support Syst..

[55] You L., Zha D., Anslyn E.V. (2015). Recent Advances in Supramolecular Analytical Chemistry Using Optical Sensing. Chem. Rev..

[56] Chaudhuri S., DiScenza D.J., Smith B., Yocum R., Levine M. (2017). Array-based detection of isomeric and analogous analytes employing synthetically modified fluorophore attached β-cyclodextrin derivatives. New J. Chem..

[57] Li Z., Suslick K.S. (2019). Colorimetric Sensor Array for Monitoring CO and Ethylene. Anal. Chem..

[58] Tropp J., Ihde M.H., Crater E.R., Bell N.C., Bhatta R., Johnson I.C., Bonizzoni M., Azoulay J.D. (2020). A Sensor Array for the Nanomolar Detection of Azo Dyes in Water. ACS Sens..

[59] Li Z., Wang Z., Khan J., Lagasse M.K., Suslick K.S. (2020). Ultrasensitive Monitoring of Museum Airborne Pollutants Using a Silver Nanoparticle Sensor Array. ACS Sens..

[60] Lin H. (2021). Rice Freshness Identification Based on Visible Near-Infrared Spectroscopy and Colorimetric Sensor Array. Food Anal. Methods.

[61] Han J., Cheng H., Wang B., Braun M.S., Fan X., Bender M., Huang W., Domhan C., Mier W., Lindner T. (2017). A Polymer/Peptide Complex-Based Sensor Array That Discriminates Bacteria in Urine. Angew. Chem. Int. Ed..

[62] Caracciolo G., Safavi-Sohi R., Malekzadeh R., Poustchi H., Vasighi M., Zenezini Chiozzi R., Capriotti A.L., Laganà A., Hajipour M., Di Domenico M. (2019). Disease-Specific Protein Corona Sensor Arrays May Have Disease Detection Capacity. Nanoscale Horiz..

[63] Yin M., Jing C., Li H., Deng Q., Wang S. (2020). Surface Chemistry Modified Upconversion Nanoparticles as Fluorescent Sensor Array for Discrimination of Foodborne Pathogenic Bacteria. J. Nanobiotechnol..

[64] Ma Y., Ai W., Huang J., Ma L., Geng Y., Liu X., Wang X., Yang Z., Wang Z. (2020). Mitochondria-Targeted Sensor Array with Aggregation-Induced Emission Luminogens for Identification of Various Cells. Anal. Chem..

[65] Shi Y., Gao X., Wei W., Chen Y. (2021). Development of Array-Based Gold Nanoclusters for Discrimination of CA125 Overexpressed Serum Samples. Adv. Mater. Sci. Eng..

[66] Tomita S., Sakao M., Kurita R., Niwa O., Yoshimoto K. (2015). A Polyion Complex Sensor Array for Markerless and Noninvasive Identification of Differentiated Mesenchymal Stem Cells from Human Adipose Tissue. Chem. Sci..

[67] Svechkarev D., Sadykov M.R., Bayles K.W., Mohs A.M. (2018). Ratiometric Fluorescent Sensor Array as a Versatile Tool for Bacterial Pathogen Identification and Analysis. ACS Sens..

[68] Levine M. (2021). Fluorescence-Based Sensing of Pesticides Using Supramolecular Chemistry. Front. Chem..

[69] El-Wahab H.M.F.A., Moram G.S.E.D. (2013). Toxic Effects of Some Synthetic Food Colorants and/or Flavor Additives on Male Rats. Toxicol. Ind. Health.

[70] Mitra R., Ayyannan S.R. (2021). Small-Molecule Inhibitors of Shp2 Phosphatase as Potential Chemotherapeutic Agents for Glioblastoma: A Minireview. ChemMedChem.

[71] Ball N., Bars R., Botham P.A., Cuciureanu A., Cronin M.T.D., Doe J.E., Dudzina T., Gant T.W., Leist M., van Ravenzwaay B. (2022). A Framework for Chemical Safety Assessment Incorporating New Approach Methodologies within REACH. Arch. Toxicol..

[72] Zhao S., Xu W., Jiang W., Yu W., Lin Y., Zhang T., Yao J., Zhou L., Zeng Y., Li H. (2010). Regulation of Cellular Metabolism by Protein Lysine Acetylation. Science.

[73] Shi L., Tu B.P. (2015). ScienceDirect Acetyl-CoA and the Regulation of Metabolism: Mechanisms and Consequences. Curr. Opin. Cell Biol..

[74] Filipiak W., Mochalski P., Filipiak A., Ager C., Cumeras R., Davis C.E., Agapiou A., Unterkofler K., Troppmair J. (2016). A Compendium of Volatile Organic Compounds (VOCs) Released by Human Cell Lines. Curr. Med. Chem..

[75] Opri O., Ildikó M.S., Dana L., Lucian C. (2019). Evaluation of the Photosynthetic Parameters, Emission of Volatile Organic Compounds and Ultrastructure of Common Green Leafy Vegetables after Exposure to Non-Steroidal Anti-Inflammatory Drugs (NSAIDs). Ecotoxicology.

[76] Pasini P., Powar N., Sylvia R.G., Aldo D. (2004). Use of a Gas-Sensor Array for Detecting Volatile Organic Compounds (VOC) in Chemically Induced Cells. Anal. Bioanal. Chem..

[77] Liu Q., Tian J., Jiang M., Qiao X., Xu Z. (2018). Direct Competitive Biomimetic Immunoassay Based on Quantum Dot Label for Simultaneous Determination of Two Pesticide Residues in Fruit and Vegetable Samples. Food Anal. Methods.

[78] Jiang M., Chattopadhyay A.N., Geng Y., Rotello V.M. (2022). An Array-Based Nanosensor for Detecting Cellular Responses in Macrophages Induced by Femtomolar Levels of Pesticides. Chem. Commun..

[79] Li J., Fan C., Pei H., Shi J., Huang Q. (2013). Smart Drug Delivery Nanocarriers with Self-Assembled DNA Nanostructures. Adv. Mater..

[80] Li Q., Liu L., Liu J., Jiang J., Yu R., Chu X. (2014). Trends in Analytical Chemistry Nanomaterial-Based Fluorescent Probes for Live-Cell Imaging. Trends Anal. Chem..

[81] Huang W., Tao F., Li F., Mortimer M., Guo L. (2020). NanoImpact Antibacterial Nanomaterials for Environmental and Consumer Product Applications. NanoImpact.

[82] Tirumala M.G., Anchi P., Raja S., Rachamalla M. (2021). Novel Methods and Approaches for Safety Evaluation of Nanoparticle Formulations: A Focus Towards In Vitro Models and Adverse Outcome Pathways. Front. Pharmacol..

[83] Shah P., Zhu X., Zhang X., He J., Li C. (2016). Microelectromechanical System-Based Sensing Arrays for Comparative in Vitro Nanotoxicity Assessment at Single Cell and Small Cell-Population Using Electrochemical Impedance Spectroscopy. ACS Appl. Mater. Interfaces.

[84] Rana S., Singla A.K., Bajaj A., Elci S.G., Miranda O.R., Mout R., Yan B., Jirik F.R., Rotello V.M. (2012). Array-Based Sensing of Metastatic Cells and Tissues Using Nanoparticle–Fluorescent Protein Conjugates. ACS Nano.

[85] Geng Y., Chattopadhyay A.N., Zhang X., Jiang M., Luther D.C., Gopalakrishnan S., Rotello V.M. (2020). Nano Assessing Nano: Nanosensor-Enabled Detection of Cell Phenotypic Changes Identifies Nanoparticle Toxicological Effects at Ultra-Low Expo-sure Levels. Small.

[86] Le N.D.B., Wang X., Geng Y., Tang R., Jiang Z., Rotello V.M. (2017). Disrupting Chemicals Using a Nanosensor-Enabled Cell-Based Platform. Chem. Commun..

[87] Dorato M.A., Buckley L.A. (2006). Toxicology in The Drug Discovery and Development Process. Curr. Protoc. Pharmacol..

[88] Perazella M.A. (2019). Drug-Induced Acute Kidney Injury: Diverse Mechanisms of Tubular Injury. Curr. Opin. Crit. Care.

[89] Yu X., Bai X., Zhang R., Zhang Y., Hu Y., Lu M., Yu B., Liu S., Tian J. (2021). A Nanosensor for Precise Discrimination of Nephrotoxic Drug Mechanisms via Dynamic Fluorescence Fingerprint Strategy. Anal. Chim. Acta.

[90] Jiang M., Wu S., Xu L., Qiao X., Xu Z. (2017). Determination of Trichlorfon Residues in Vegetables Using a Quantum Dot-Labeled Biomimetic Immunoassay Method Followed by Capillary Electrophoresis. Food Agric. Immunol..

[91] Markowicz M., Mikiciuk-olasik E. (2012). Adaptation of High-Throughput Screening in Drug Discovery—Toxicological Screening Tests. Int. J. Mol. Sci..

[92] Butcher R.A., Schreiber S.L. (2005). Using Genome-Wide Transcriptional Profiling to Elucidate Small-Molecule Mechanism. Curr. Opin. Chem. Biol..

[93] Ma S., Morrison R., Hobbs S.J., Soni V., Farrow-johnson J., Frando A., Fleck N., Grundner C., Rhee K.Y., Rustad T.R. (2021). Transcriptional Regulator-Induced Phenotype Screen Reveals Drug Potentiators in Mycobacterium Tuberculosis. Nat. Microbiol..

[94] Abildgaard C., Rizza S., Christiansen H., Schmidt S., Dahl C., Al A.A., Christensen A., Filomeni G. (2021). Screening of Metabolic Modulators Identifies New Strategies to Target Metabolic Reprogramming in Melanoma. Sci. Rep..

[95] Rana S., Le N.D.B., Mout R., Saha K., Tonga G.Y., Bain R.E.S., Miranda O.R., Rotello C.M., Rotello V.M. (2015). A Multichannel Nanosensor for Instantaneous Readout of Cancer Drug Mechanisms. Nat. Nanotechnol..

[96] Xiang S., Nie F., Zhang C. (2008). Learning a Mahalanobis Distance Metric for Data Clustering and Classification. Pattern Recognit..

[97] Geng Y., Amante J.J., Goel H.L., Zhang X., Walker M.R., Luther D.C., Mercurio A.M., Rotello V.M. (2020). Differentiation of Cancer Stem Cells through Nanoparticle Surface Engineering. ACS Nano.

[98] Muppidathi M., Perumal P., Ayyanu R., Subramanian S. (2019). Immobilization of SsDNA on a Metal—Organic Framework Derived Magnetic Porous Carbon (MPC) Composite as a Fluorescent Sensing Platform for the Detection of Arsenate Ions. Analyst.

[99] Das Saha N., Sasmal R., Meethal S.K., Vats S., Gopinathan P.V., Jash O., Manjithaya R., Gagey-Eilstein N., Agasti S.S. (2019). Multichannel DNA Sensor Array Fingerprints Cell States and Identifies Pharmacological Effectors of Catabolic Processes. ACS Sens..

[100] Xing Y., Zhao L., Cheng Z., Lv C., Yu F., Yu F. (2021). Micro Fluidics-Based Sensing of Biospecies. ACS Appl. Bio Mater..

[101] Sugai H., Tomita S., Ishihara S., Yoshioka K., Kurita R. (2020). Microfluidic Sensing System with a Multichannel Surface Plasmon Resonance Chip: Damage-Free Characterization of Cells by Pattern Recognition. Anal. Chem..

